# Eco-Friendly Synthesis of Zirconia Nanoparticles Using *Sonchus asper* Extract: A Sustainable Approach to Enhancing Chinese Cabbage Growth and Remediating Chromium-Contaminated Soil

**DOI:** 10.3390/toxics13050324

**Published:** 2025-04-22

**Authors:** Guojie Weng, Weidong Li, Fengyue Qin, Menglu Dong, Shuangqi Yue, Sajid Mehmood, Xu Wang

**Affiliations:** 1 Center for Eco-Environment Restoration of Hainan Province, School of Ecology, Hainan University, Haikou 570228, China; 2 School of Topical Agriculture and Foresty, Hainan University, Haikou 570228, China

**Keywords:** zirconia nanoparticles, chromium remediation, soil nutrient retention, oxidative stress, antioxidant enzymes

## Abstract

Chromium (Cr) contamination poses severe risks to plant health and soil quality, requiring sustainable remediation methods. This study explored the synthesis of zirconia nanoparticles (PF-ZrO_2_ NPs) from *Sonchus asper* extract and assessed their potential to alleviate Cr toxicity in Chinese cabbage (*Brassica rapa* var. pekinensis). The characterization of nanoparticles was performed through XRD, SEM, and FTIR analyses, confirming their crystalline nature, structure, and surface chemistry. The results indicated significant declines in plant growth, chlorophyll content, biomass, and nutrient uptake under Cr stress (treatments T2 and T4), accompanied by elevated oxidative stress indicators (H_2_O_2_, MDA) and Cr accumulation. The application of PF-ZrO_2_ NPs (T3 and T5) notably reduced shoot Cr concentrations (by 58.94% and 35.90%) and improved the chlorophyll level (by 5.41% and 14.41%). Additionally, nanoparticles increased antioxidant enzyme activity (SOD, POD, CAT) and improved soil properties (pH, cation exchange capacity, nutrient retention). These findings suggest green-synthesized PF-ZrO_2_ NPs are effective, environmentally friendly candidates for Cr remediation in contaminated soils.

## 1. Introduction

Soil contamination with heavy metals, particularly chromium (Cr), is a pressing environmental issue that severely affects soil fertility, plant productivity, and food safety [1]. Chromium pollution arises from industrial activities such as metal plating, tannery operations, textile dyeing, and mining, leading to its accumulation in agricultural lands [2]. Trivalent chromium (Cr (III)) and hexavalent chromium (Cr (VI)) are the two primary oxidation states of chromium found in soil [3,4]. Cr (III) is considered less toxic and, in trace amounts, essential for some biological functions, while Cr (VI) is highly toxic and carcinogenic [5,6]. Additionally, Cr (VI) is water-soluble, which increases its bioavailability and makes it more hazardous to living organisms [3,4]. Chromium (Cr) toxicity in plants results in nutrient imbalance, root injury, and leaf chlorosis, and it inhibits chlorophyll biosynthesis [7]. Excessive Cr uptake leads to the generation of reactive oxygen species (ROS), causing oxidative stress, which impairs plant growth and essential metabolic processes [8]. Additionally, Cr contamination reduces crop yield and poses risks to human health through food chain contamination [9].

Traditional approaches for remediating heavy-metal-contaminated soils include chemical leaching, soil washing, electrokinetic remediation, and phytoremediation [10]. While effective, these methods are often costly, labor-intensive, and environmentally disruptive, sometimes leading to secondary pollution. Alternatively, the application of nanotechnology in environmental remediation has gained attention due to the high surface reactivity, adsorption capacity, and tunable properties of nanoparticles (NPs) [11,12]. Among various nanomaterials, zirconium-based nanoparticles have shown potential in immobilizing heavy metals due to their strong affinity for metal ions and stability under varying environmental conditions [13,14,15,16]. Nanoparticles, due to their unique surface properties and small size, have shown great potential in the remediation of contaminated soils and water. They can absorb/adsorb a variety of contaminants and catalyze reactions by lowering the energy required for breakdown [17,18]. The nanoscale size of particles increases their surface area, which enhances their reactivity and adsorption capacity, making them more effective for contaminant removal [19].

Recent green synthesis approaches have gained significant attention for producing zirconia nanoparticles due to their eco-friendly nature and cost-effectiveness [20,21,22]. Plant extracts have emerged as a promising option for the biosynthesis of nanoparticles, aligning with the United Nations’ sustainability agenda 2030 [23]. These methods offer advantages such as non-toxicity, higher stability, and superior performance compared to conventional physicochemical approaches. *Eucalyptus globulus* extract and its essential oils have been successfully utilized to fabricate various nanoparticles, including zirconia oxide [23]. This green synthesis method adheres to environmentally benign routes and offers better control over the size and shape of the nanoparticles. Interestingly, while Sadiq et al. (2023) [23] focus on plant-based synthesis, Kumari et al. (2022) [24] mention that zirconia nanoparticles can also be prepared using bacteria and fungi, highlighting the versatility of green synthesis approaches. This method eliminates the need for hazardous chemicals, reduces production costs, and enhances biocompatibility, making it an attractive alternative for large-scale applications. It minimizes hazardous waste, operates under milder conditions (e.g., ambient temperature and pressure), and utilizes renewable, biodegradable resources. This approach is not only cost-effective but also aligns with the principles of green chemistry, making it more sustainable and suitable for large-scale and environmentally conscious applications. *Sonchus asper*, commonly known as prickly sow-thistle, is a medicinal plant rich in bioactive compounds such as flavonoids, polyphenols, and alkaloids, which exhibit strong antioxidant and metal-chelating properties [25,26]. These phytochemicals facilitate the biosynthesis of metal-based nanoparticles while enhancing their stability and functional properties.

Chinese cabbage (*Brassica rapa* var. pekinensis), a widely cultivated leafy vegetable, is highly sensitive to Cr-induced stress, which adversely affects its growth, nutrient uptake, and physiological processes [27]. When exposed to Cr-contaminated soil, cabbage plants experience increased oxidative stress, reduced chlorophyll content, stunted growth, and lower biomass production [27]. Recent studies suggest that nanomaterials can mitigate heavy metal toxicity in plants by adsorbing metal ions, reducing their bioavailability, and enhancing plant tolerance through improved antioxidant defense mechanisms [28].

This study aims to evaluate the efficacy of ZrO_2_ nanoparticles synthesized using *Sonchus asper* extract in remediating Cr-contaminated soil while promoting the healthy growth of Chinese cabbage. We hypothesize that the application of these nanoparticles will (i) enhance plant growth parameters, including biomass, chlorophyll content, and antioxidant enzyme activity, and (ii) reduce Cr bioavailability in soil, leading to improved soil quality and crop productivity. By integrating nanotechnology with plant-based green chemistry, this research contributes to the development of a cost-effective, sustainable, and environmentally safe solution for heavy metal remediation in agricultural systems.

## 2. Materials and Methods

### 2.1. Chemicals and Materials

Zirconium oxychloride octahydrate (ZrOCl_2_·8H_2_O, 98.99%) was purchased from Xilong Science Co., Ltd. (Shantou, China). Sulfuric acid and sodium hydroxide were provided by Shanghai Macklin Biochemical Co., Ltd. (Shanghai, China). 1,5-Diphenylcarbazide (DPC) and potassium dichromate (K_2_Cr_2_O₇) were obtained from Guangzhou Huada Chemical Reagent Co., Ltd. The absorbance of Cr (VI) was measured using a Shimadzu UV-160 spectrophotometer (Shimadzu Corporation, Kyoto, Japan). *Sonchus asper* was collected from natural farmland in Zhejiang, China.

### 2.2. Synthesis of Plant-Fabricated Zirconia Nanoparticles (PF-ZrO_2_ NPs)

*Sonchus asper* was collected from the field between October and February and washed thoroughly with distilled water to remove residual soil and decayed leaves. The plant material was air-dried and then pulverized into fine powder using a grinder (FGZ-1000C, Dongguan Fangtai Electric Appliance Co., Ltd., Dongguan, China) and it was sieved to pass through a 200-mesh screen. Approximately 10 g of the powder were mixed with 200 mL of ultrapure water and stirred at 80 ± 2 °C in a water bath for 30 min. After cooling to room temperature, the mixture was filtered to obtain a brown extract, which was used as a reducing and capping agent. Zirconium oxychloride octahydrate (0.1 M) was mixed with the extract in a 1:1 (*v*/*v*) ratio and heated at 70 °C for 6 h. The formation of a white precipitate suggests the successful synthesis of ZrO_2_ nanoparticles, which aligns with the characteristics of zirconia described in the literature. For instance, Kazemi et al. (2020) [29] discuss the synthesis of nano-zirconia using zirconium hydroxide as a metal precursor, followed by calcination at various temperatures. The use of zirconium oxychloride octahydrate in the given process is comparable to this approach. The mixture was then centrifuged at 5000 rpm for 10 min to obtain a brown precipitate, which was dried in an oven at 70 °C. The dried sample was subsequently heated in a muffle furnace at 500 °C for 4 h to produce pure PF-ZrO_2_ NPs.

### 2.3. Characterization of PF-ZrO_2_ Nanoparticles

A comprehensive characterization of plant-fabricated zirconia nanoparticles (PF-ZrO_2_ NPs) was performed using multiple advanced analytical instruments. X-ray diffraction (XRD) analysis was conducted with a Rigaku Smart Lab X-ray diffractometer (Tokyo, Japan) to determine the crystal structure and phase composition of PF-ZrO_2_ NPs. XRD patterns confirmed the presence of zirconia crystal phases and provided foundational information for qualitative analysis. The surface morphology and microstructure were observed in detail using a Thermo Fisher Verios G4 UC scanning electron microscope (SEM) (Czech Republic), allowing precise visualization of particle shape, size distribution, and aggregation behavior. SEM analysis has revealed important information about particle size, shape, and agglomeration in different materials. For instance, in B4C/Al composites, SEM showed that larger B4C particle sizes exhibited a more uniform and discrete distribution, while smaller sizes tended to agglomerate [30]. Similarly, SEM micrographs of Ag/AgX photocatalysts displayed agglomeration with non-uniform particle distribution, which is typical of surfactant-free precipitation reactions in aqueous media [31]. To further investigate the internal structure, a Thermo Scientific Talos F200X G2 (Waltham, MA, USA) transmission electron microscope (TEM) (Czech Republic) was employed. High-resolution TEM imaging allowed for the examination of the crystal structure, particle size, morphology, and lattice interactions of PF-ZrO_2_ NPs. Fourier-transform infrared spectroscopy (FTIR) analysis was conducted using a Thermo Nicolet iS50 spectrometer (Waltham, MA, USA) to identify functional groups on the surface of PF-ZrO_2_ NPs.

The specific surface area, pore volume, and pore size distribution of the green-synthesized plant-derived zirconia (PF-ZrO_2_) nanoparticles were analyzed using Brunauer–Emmett–Teller (BET) and Barrett-Joyner-Halenda (BJH) methods. The nitrogen adsorption–desorption isotherms of PF-ZrO_2_ NPs were recorded using a BET surface area analyzer (Micromeritics ASAP 2020, Micromeritics Instrument Corporation, Norcross, GA, USA). Before analysis, the samples were degassed under a vacuum at 150 °C for 4 h to remove any adsorbed moisture or impurities. The BET surface area was determined by analyzing the adsorption data in the relative pressure (P/P_0_) range of 0.05–0.30, assuming multilayer adsorption of gas molecules on the solid surface.

### 2.4. Pore Size Distribution and Pore Volume Analysis

The pore size distribution and total pore volume were determined using the Barrett-Joyner-Halenda (BJH) method from the desorption branch of the isotherm. The data were obtained by applying the BJH algorithm to the adsorption–desorption isotherm, with the pore size range analyzed to be between 2 and 60 nm to confirm the presence of mesopores. The pore volume was calculated at P/P_0_ = 0.99, representing the total nitrogen uptake at near-saturation conditions.

### 2.5. Plant Growth Experiment

#### 2.5.1. Experimental Design and Setup

A pot experiment was conducted to evaluate the effects of plant-derived zirconia nanoparticles (PF-ZrO_2_ NPs) on the growth and physiological response of Chinese cabbage (*Brassica rapa* var. pekinensis) under chromium (Cr) stress. The experiment aimed to assess the potential of these nanoparticles in enhancing plant health and reducing Cr bioavailability in contaminated soil.

#### 2.5.2. Soil Collection and Preparation

The soil samples used in this study were collected, and initial physicochemical properties of the collected soil was comprehensively analyzed by Suzhou Grace Biotechnology Co., Ltd., Suzhou, China. Specifically, the soil pH was determined using a direct measurement method with a pH meter, and the initial pH of the collected soil was recorded as approximately 6.34. The electrical conductivity (EC) was measured using an electrical conductivity meter, and the initial EC was determined to be approximately 6.8 mS/cm. The chromium (Cr) content was analyzed using strong acid digestion followed by atomic absorption spectroscopy (AAS), with the initial Cr concentration found to be approximately 0.131–0.134 mg/kg, indicating minimal contamination levels. Following collection and initial characterization, the soil was artificially contaminated by adding potassium dichromate (K_2_Cr_2_O₇) solutions to achieve final Cr (VI) concentrations of 50 mg/kg and 100 mg/kg. The spiked soils were thoroughly homogenized, maintained at optimal moisture levels (approximately 70% water-holding capacity), and incubated for two months to ensure equilibrium and simulate realistic environmental conditions before use in the subsequent plant growth experiments.

#### 2.5.3. Seed Sowing and Nanoparticle Application

Chinese cabbage seeds were surface sterilized using 0.1% sodium hypochlorite (NaOCl) for 5 min, followed by multiple washes with distilled water. The seeds were then sown directly into pots containing Cr-contaminated soil. To evaluate the effect of plant-derived zirconia nanoparticles (PF-ZrO_2_ NPs) on plant growth and chromium remediation, the following treatment conditions were applied: T1: 0 mg/kg Cr + 0 mg/kg PF-ZrO_2_ NPs (Control); T2: 50 mg/kg Cr; T3: 50 mg/kg Cr + 500 mg/kg PF-ZrO_2_ NPs; T4: 100 mg/kg Cr; and T5: 100 mg/kg Cr + 500 mg/kg PF-ZrO_2_ NPs. The chromium dosage was selected based on the study by Antoniadis et al. (2018) [32], while the PF-ZrO_2_ NPs dosage was adapted from Wang et al. (2009) [33] to ensure consistency with previously established methodologies. Each treatment was replicated three times in a completely randomized design (CRD). The pots were maintained under greenhouse conditions with a day/night temperature of 25/18 °C, relative humidity of 65%, and photoperiod of 14 h light/10 h dark. Plants were irrigated with deionized water at regular intervals to maintain field capacity moisture.

### 2.6. Sample Collections

At the end of the experiment, the roots and shoots of the plants and the soil in the pots were collected. The roots of the plants were washed with deionized water to remove the PF-ZrO_2_ NPs attached and air-dried for moisture calculation. The length and weight of each plant’s root and shoot were measured. The samples were stored at −80 °C for further analysis. Chromium content, photosynthetic pigment content, antioxidant enzyme system activity (SOD, POD, CAT, H_2_O_2_, and MDA), soluble protein and soluble sugar content, proline content, and nutrient element (N, P, K, Ca, Mg) content were determined in the tissues of the root and shoot, respectively.

### 2.7. Methods of Analysis

The total chromium (Cr) content in soil and plant samples was quantified using graphite furnace atomic absorption spectrometry (GFAAS) at 357.9 nm. Samples were digested using appropriate acid mixtures based on matrix type, followed by filtration and instrumental analysis. The principle of the method is to determine the Cr content in food stuffs by using graphite furnace atomic absorption spectrometry at 357.9 nm after the sample is digested, and the absorption value is compared with the standard series of solutions to quantify the Cr content in a certain concentration range. Chlorophyll content, SOD, POD, CAT, H_2_O_2_, MDA, soluble protein, soluble sugar, and proline content were determined using kits from Suzhou Grace Biotechnology Co., Suzhou, China. The results were determined according to the following methods: Chlorophyll content determination kit (G0601W), Superoxide dismutase (SOD)-WST-8 method activity determination kit (G0101W), Peroxidase (POD) kit (G0107W), Catalase (CAT) activity kit (G0105W), Hydrogen peroxide content (H_2_O_2_) determination kit (G0168W), Malondialdehyde (MDA) content kit (G0109W), Kaumas Brilliant Blue assay for protein content determination kit (G0417W), Soluble Sugar content kit (G0501W), and Proline (PRO) content determination kit (G0111W). Carotenoids were determined by high performance liquid chromatography (HPLC) using Shimadzu LC-20A chromatograph, and nutrient contents were determined by atomic absorption method. PH was determined by potentiometric method, pH meter HJ 962-2018. The electrode method was used to determine EL using a DDS-11A type conductivity meter.

### 2.8. Soil Physicochemical Properties After the Experiment

To determine the impact of PF-ZrO_2_ NPs on soil characteristics and their role in chromium remediation, the key soil parameters were analyzed post-experiment.

The soil pH was measured in a 1:2.5 soil-to-water suspension using a digital pH meter (Hanna Instruments HI 2211, Woonsocket, RI, USA). The EC was measured in a 1:5 soil-to-water suspension using an EC meter (EUTECH CON 700, Thermo Scientific, Waltham, MA, USA) at 25 °C, with values expressed in dS/m. The CEC was determined using the 1M ammonium acetate method (NH_4_OAc, pH 7.0), and exchangeable cations (Ca^2^⁺, Mg^2^⁺, K⁺) were quantified using AAS.

### 2.9. Data Analysis

Validation of data was conducted to ensure the reliability and accuracy of analytical procedures utilized in this study. Precision was determined through repeated analyses (*n* = 3) of standard samples, resulting in relative standard deviations (RSD%) less than 5%, confirming the method’s high precision. These validation parameters collectively demonstrate that the analytical methods employed in this study are accurate, precise, and robust for assessing chromium content in soil and plant samples.

All the collected data were statistically analyzed using one-way ANOVA to determine the significance of nanoparticle treatments on plant growth, biochemical responses, and chromium accumulation. Tukey’s HSD test (*p* < 0.05) was used for multiple comparisons among treatments [34]. Statistical analyses were performed using IBM SPSS Statistics 25.

## 3. Results and Discussion

### 3.1. Morphological and Structural Characterization of PF-ZrO_2_ Nanoparticles

**Figure 1:**
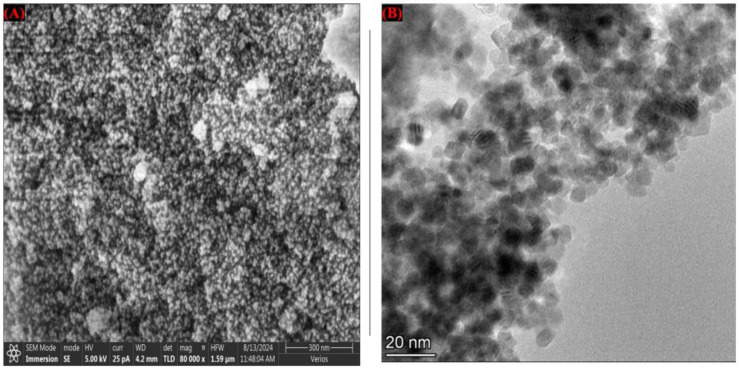
(**A**) Scanning electron microscopy (SEM) image of green-synthesized PF-ZrO_2_ nanoparticles. (**B**) Transmission electron microscopy (TEM) image of PF-ZrO_2_ NPs.

The morphology, size, and surface characteristics of the green-synthesized plant-derived zirconia (PF-ZrO_2_) nanoparticles were examined using scanning electron microscopy (SEM) and transmission electron microscopy (TEM) (Figure 1). The SEM image (Figure 1A) revealed that PF-ZrO_2_ NPs exhibited a porous structure, indicating their high surface area and potential for enhanced adsorption of heavy metals [35]. The nanoparticles appeared to be uniformly distributed, suggesting that the green synthesis method facilitated the formation of well-dispersed particles [36,37,38].

The TEM image (Figure 1B) provided further insights into the nanoparticle size and shape. The nanoparticles displayed a spherical to slightly polyhedral morphology with an average size in the nanometer range (~20 nm). The clear and well-defined edges observed in the TEM micrograph indicate the crystalline nature of the nanoparticles [39], which was further confirmed by X-ray diffraction (XRD) analysis. The nanoscale dimensions and uniform morphology suggest that PF-ZrO_2_ NPs possess enhanced reactivity and adsorption potential, making them effective for heavy metal immobilization in contaminated soils. The small particle size and high surface area of PF-ZrO_2_ NPs contribute to their high adsorption capacity and reactivity [40]. These properties play a crucial role in reducing Cr bioavailability and improving plant growth, as observed in the present study. The biogenic synthesis approach using *Sonchus asper* extract may have contributed to the uniform dispersion and stabilization of nanoparticles, as plant-derived biomolecules act as capping and reducing agents, preventing excessive particle aggregation [41,42,43].

### 3.2. Fourier-Transform Infrared Spectroscopy (FTIR) Analysis

**Figure 2:**
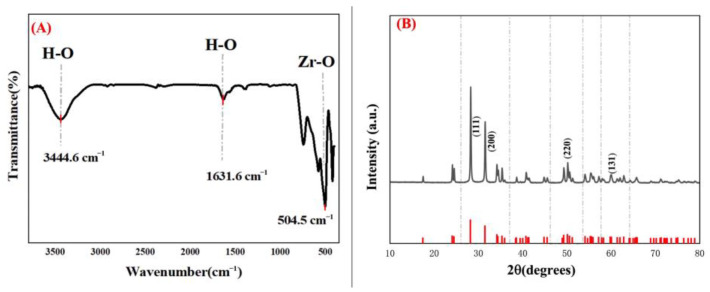
(**A**) Fourier-transform infrared spectroscopy (FTIR) spectrum of PF-ZrO_2_ nanoparticles, showing key functional groups, including hydroxyl (-OH) and zirconium-oxygen (Zr-O) bonds. (**B**) X-ray diffraction (XRD) pattern of PF-ZrO_2_ NPs, confirming their crystalline nature and phase purity.

The FTIR spectrum of the green-synthesized PF-ZrO_2_ nanoparticles (Figure 2A) confirmed the presence of key functional groups associated with the synthesis process. The broad absorption peak at 3444.6 cm^−1^ corresponds to the O-H stretching vibration of hydroxyl groups, indicating the presence of water molecules or surface-bound hydroxyls, which may have originated from plant-derived biomolecules during the synthesis [44,45]. Another peak at 1631.6 cm^−1^ corresponds to H-O bending vibrations, further confirming the presence of moisture or organic residues from the plant extract [46].

A strong absorption band at 504.5 cm^−1^ was assigned to Zr-O stretching vibrations, confirming the successful formation of zirconia nanoparticles [47]. The absence of additional strong peaks in the fingerprint region indicates minimal interference from organic impurities, suggesting that the green synthesis method produced relatively pure ZrO_2_ nanoparticles. The presence of biomolecules from plant extracts, such as phenolic compounds, flavonoids, and other antioxidants, act as reducing and capping agents during nanoparticle formation [48,49]. These biomolecules contribute to the stability and reactivity of the nanoparticles through the presence of functional groups like hydroxyl and carboxyl moieties on their surfaces.

### 3.3. X-Ray Diffraction (XRD) Analysis

The XRD pattern of PF-ZrO_2_ nanoparticles (Figure 2B) further confirmed their crystalline nature. The distinct diffraction peaks at 2θ values of approximately 30.2°, 35.2°, 50.3°, and 60.1° correspond to the (111), (200), (220), and (311) crystal planes, respectively, which match the standard reference pattern for zirconium oxide [50]. The sharp and well-defined peaks indicate high crystallinity [51], confirming the formation of well-structured nanoparticles.

The high-intensity peak at 30.2° (111) suggests that ZrO_2_ nanoparticles possess a face-centered cubic (FCC) structure, which is known for its stability and catalytic efficiency. The absence of additional impurity peaks indicates the successful synthesis of phase-pure ZrO_2_ nanoparticles without unwanted secondary phases [52]. The crystallite size, estimated using the Scherrer equation, was found to be in the nanometer range (~20 nm), which is consistent with the TEM observations.

### 3.4. Energy-Dispersive X-Ray Spectroscopy (EDS) and Elemental Mapping Analysis

The elemental composition and spatial distribution of elements in the green-synthesized PF-ZrO_2_ nanoparticles were analyzed using energy-dispersive X-ray spectroscopy (EDS) and elemental mapping (Figure S1A,B). The EDS spectrum (Figure S1A) confirmed the presence of zirconium (Zr), oxygen (O), and carbon (C) as the primary elements in the synthesized nanoparticles. The quantitative analysis showed that Zr was the dominant element (53.5%), followed by O (28.9%) and C (17.6%), indicating the formation of zirconia nanoparticles [53]. The presence of oxygen suggests the formation of oxidation states related to ZrO_2_, while carbon likely originated from plant-derived organic compounds used during the green synthesis process [54,55]. The elemental mapping images (Figure S1B) further validated the uniform distribution of Zr, O, and C throughout the nanoparticles. The Zr signals were well-distributed, confirming the homogeneity of the synthesized NPs, while the O and C signals demonstrated the successful interaction of plant-derived biomolecules with Zr, contributing to nanoparticle stabilization. The uniformity in elemental distribution suggests that the green synthesis method resulted in well-structured and stable nanoparticles, which are essential for their high adsorption capacity and efficiency in Cr immobilization. The high Zr content, along with its well-defined distribution, suggests that PF-ZrO_2_ NPs are highly efficient in binding heavy metals, making them suitable for soil remediation applications [56].

### 3.5. Surface Area and Pore Structure Analysis of PF-ZrO_2_ Nanoparticles

**Figure 3:**
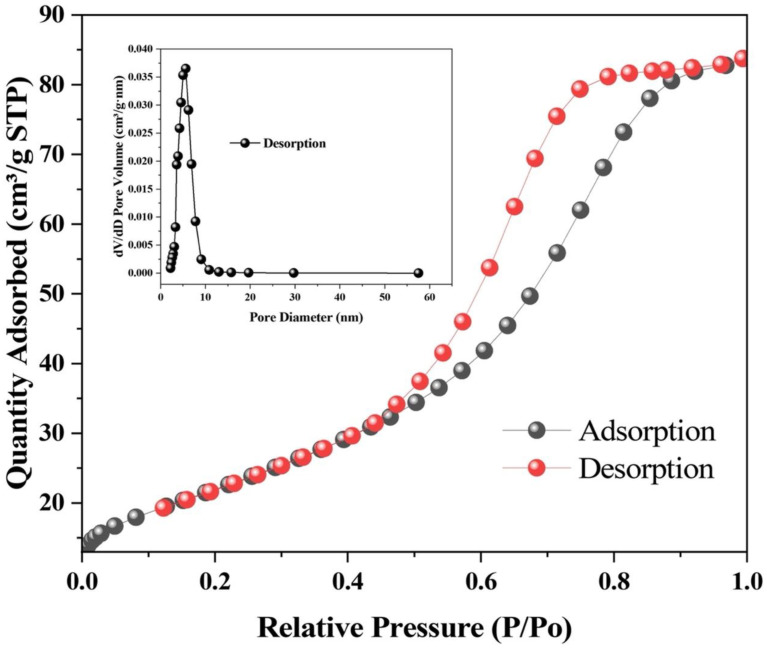
N_2_ adsorption–desorption isotherms of PF-ZrO_2_ nanoparticles. The isotherm shows a Type IV pattern with a Type H2(a) hysteresis loop. The inset graph shows the pore size distribution (BJH method).

The specific surface area, pore size, and adsorption–desorption behavior of the green-synthesized PF-ZrO_2_ nanoparticles were analyzed using the Brunauer-Emmett-Teller (BET) isotherm and Barrett-Joyner-Halenda (BJH) pore size distribution methods (Figure 3). The N_2_ adsorption–desorption isotherm exhibits a Type IV isotherm with a Type H2(a) hysteresis loop, as defined by IUPAC guidelines [57], suggesting the presence of complex pore structures with pore-blocking effects. The adsorption branch shows a gradual increase in nitrogen uptake at low relative pressure (P/P_0_ < 0.3), indicating monolayer adsorption, followed by a steep increase at higher P/P_0_ values, suggesting multilayer adsorption and capillary condensation in mesopores [58,59].

The inset pore size distribution plot (BJH method) confirms that PF-ZrO_2_ nanoparticles possess a narrow mesoporous structure, with the majority of pore diameters falling below 10 nm. The high surface area and well-defined mesoporosity of PF-ZrO_2_ NPs are beneficial for adsorption applications, as they enhance the nanoparticles’ ability to capture heavy metal ions, including Cr (VI), from contaminated soils. The presence of mesopores facilitates Cr immobilization by providing abundant active sites for adsorption and interaction with soil components, thereby reducing Cr bioavailability and plant uptake [60].

### 3.6. Soil Physicochemical Properties Under Chromium Stress and PF-ZrO_2_ NP Application

**Figure 4:**
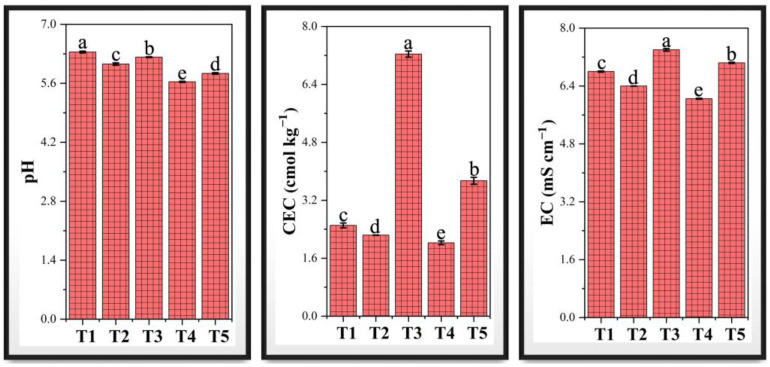
Effect of chromium (Cr) stress and PF-ZrO_2_ NP application on soil pH, cation exchange capacity (CEC), and electrical conductivity (EC). T1: Control (0 mg/kg Cr + 0 mg/kg PF-ZrO_2_ NPs), T2: 50 mg/kg Cr, T3: 50 mg/kg Cr + 500 mg/kg PF-ZrO_2_ NPs, T4: 100 mg/kg Cr, T5: 100 mg/kg Cr + 500 mg/kg PF-ZrO_2_ NPs. Results are the mean values ± standard deviation (*n* = 3). Error bars indicate standard deviations. Different small letters on the bars indicate significant differences among treatments at *p* < 0.05.

The pH, electrical conductivity (EC), and cation exchange capacity (CEC) of soil were significantly affected by chromium stress and PF-ZrO_2_ NP application (Figure 4). Chromium contamination resulted in significant soil acidification, as evidenced by the decrease in soil pH from 6.34 in the control (T1) to 6.06 in T2 and further to 5.64 in T4. The observed pH decline is likely due to Cr (VI) reduction and the subsequent release of H⁺ ions, which increases soil acidity, as demonstrated in the study in which using *Tamarindus indica* methanol leaves extract as a reductant [61]. A lower pH can enhance Cr solubility, increasing its bioavailability and toxicity to plants [62]. However, the application of PF-ZrO_2_ NPs in treatments T3 and T5 partially alleviated soil acidification, with pH levels increasing from 6.06 to 6.22 in T3 (compared to T2) and from 5.64 to 5.83 in T5 (compared to T4). This suggests that nanoparticles helped buffer soil acidity, reducing Cr solubility and potentially lowering its toxicity [63].

Soil EC increased under chromium stress, with T2 and T4 showing 5.88% and 11.03% higher EC values than T1, indicating enhanced ionic strength due to Cr contamination. Excessive EC can disrupt soil microbial activity and plant nutrient uptake, leading to osmotic stress [64]. However, T3 and T5 exhibited 15.62% and 16.36% reductions in EC compared to T2 and T4, respectively, suggesting that PF-ZrO_2_ NPs helped regulate ionic imbalances, possibly by adsorbing excess Cr ions and stabilizing soil salinity [65].

The most significant impact was observed on CEC, which declined by 10.77% in T2 and 19.17% in T4 compared to the control (T1), reflecting the negative effect of Cr on soil structure and its ability to retain essential nutrients. The reduction in CEC under Cr stress indicates a loss of exchangeable cations due to Cr interference with soil colloids [66]. However, the addition of PF-ZrO_2_ NPs in treatments T3 and T5 resulted in a substantial increase in cation exchange capacity (CEC), with a 223.54% rise in T3 compared to T2 and an 84.53% increase in T5 relative to T4, indicating that nanoparticles improved soil quality by enhancing cation retention, improving nutrient availability, and stabilizing soil aggregates [67]. This suggests that PF-ZrO_2_ NPs may play a crucial role in improving soil fertility in contaminated environments, making them a promising amendment for Cr remediation.

### 3.7. Macro and Micronutrient Availability in Soil Under Chromium Stress and PF-ZrO_2_ NP Application

The levels of nitrogen (N), phosphorus (P), potassium (K), calcium (Ca), and magnesium (Mg) in the soil were significantly affected by chromium contamination and PF-ZrO_2_ NP treatment (Figure S2). Chromium stress (T2 and T4) led to a 33.63% and 46.27% reduction in nitrogen content, respectively, compared to the control (T1). Similarly, phosphorus levels decreased by 24.33% in T2 and 37.53% in T4, while potassium content dropped by 54.14% and 63.74%, respectively. These findings suggest that chromium contamination disrupts nutrient availability, likely due to Cr interference with soil cation exchange processes and competitive uptake inhibition [68]. The decline in Ca (12.55% in T2, 17.58% in T4) and Mg (18.41% in T2, 23.47% in T4) further confirms Cr-induced nutrient depletion, which negatively impacts soil fertility and plant nutrition. In line with our study, Yang et al. (2021) [69] report that under aluminum stress, magnesium (Mg) content in red clover decreased significantly by 6.3% to 39.2%. This supports the general idea that metal stress can lead to nutrient depletion.

However, the application of PF-ZrO_2_ NPs (T3 and T5) significantly improved nutrient retention in soil compared to Cr-only treatments. T3 exhibited a 30.25% increase in nitrogen, a 98.56% increase in phosphorus, and a 64.26% increase in potassium compared to T2, demonstrating the nanoparticles’ potential to restore soil nutrient balance. Similarly, T5 showed a 27.81% increase in nitrogen, a 100.41% increase in phosphorus, and a 73.51% increase in potassium relative to T4, confirming the role of PF-ZrO_2_ NPs in mitigating Cr-induced nutrient depletion. Improvements in Ca (8.74% in T3, 6.66% in T5) and Mg (15.79% in T3, 19.48% in T5) suggest that the nanoparticles enhanced nutrient retention and stabilized soil fertility, creating a more favorable environment for plant growth. The positive impact of PF-ZrO_2_ NPs on nutrient retention can be attributed to their ability to immobilize Cr, reduce metal-induced nutrient displacement, and improve soil cation exchange capacity [63]. By enhancing soil structure and nutrient-holding capacity, PF-ZrO_2_ NPs promote better plant nutrient availability and uptake, ultimately leading to improved plant health and growth [70].

### 3.8. Chromium Accumulation in Soil Under PF-ZrO_2_ NP Application

**Figure 5:**
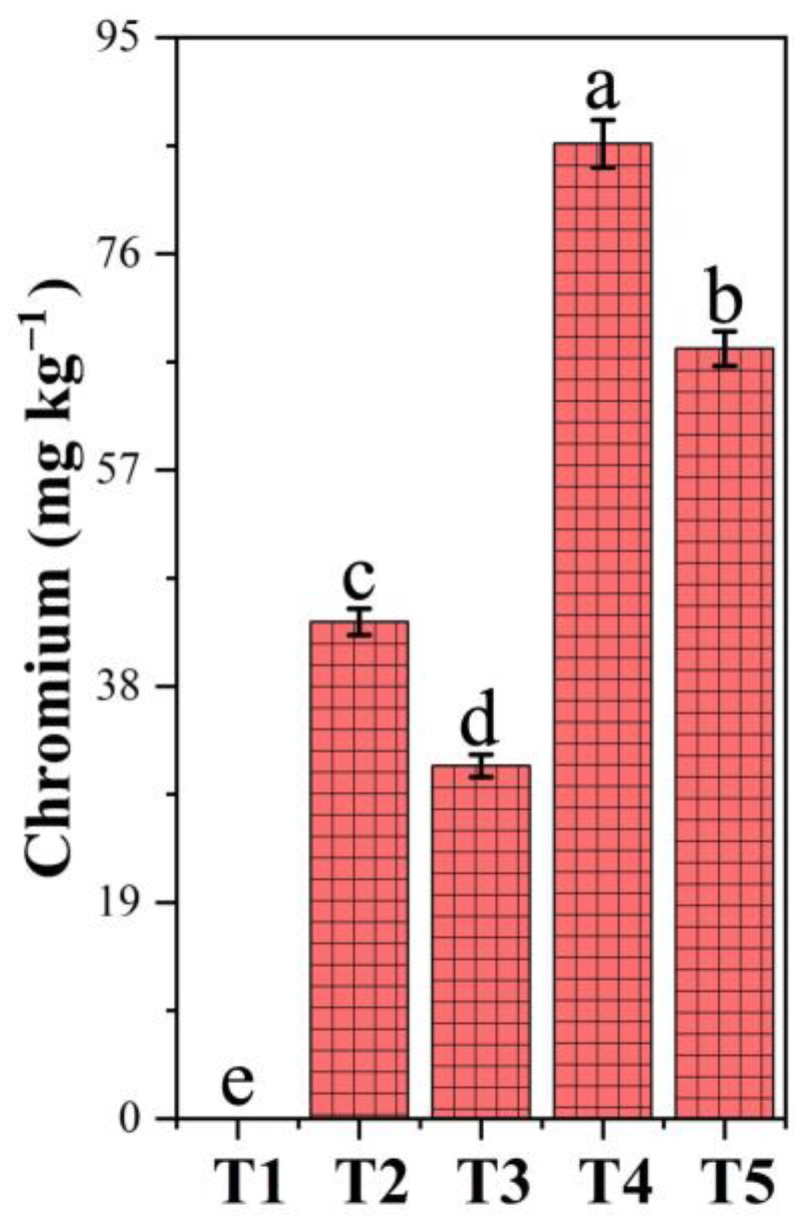
Chromium (Cr) accumulation in soil under different treatments. Error bars indicate standard deviations. Different small letters on the bars indicate significant differences among treatments at *p* < 0.05.

The chromium (Cr) accumulation in soil was significantly influenced by Cr contamination and PF-ZrO_2_ NP application (Figure 5). Chromium levels increased drastically in Cr-treated soils (T2 and T4) compared to the control (T1), with values reaching 43.67 mg/kg and 85.67 mg/kg, respectively. However, the application of PF-ZrO_2_ NPs (T3 and T5) resulted in a significant reduction in Cr. Compared to T2, T3 exhibited a 29.01% decrease in Cr concentration, while T5 showed a 21.01% reduction compared to T4. This suggests that PF-ZrO_2_ NPs effectively reduced Cr mobility and potential bioavailability, likely by adsorbing and immobilizing Cr ions in the soil, preventing them from leaching or being taken up by plants [71]. The higher reduction in T3 compared to T5 indicates that PF-ZrO_2_ NPs were more effective at lower Cr concentrations, possibly due to a higher adsorption efficiency in less saturated conditions [72]. The ability of PF-ZrO_2_ NPs to immobilize leachable Cr fractions suggests their potential as a sustainable remediation strategy for Cr-contaminated soils, through the reduction of Cr migration risks and by limiting its uptake by plants.

### 3.9. Photosynthetic Pigments Under Chromium Stress and PF-ZrO_2_ NP Application

**Figure 6:**
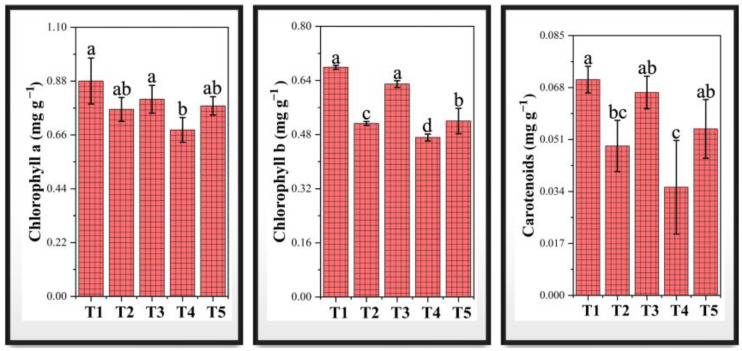
Effect of chromium (Cr) stress and PF-ZrO_2_ NP application on chlorophyll a, chlorophyll b, and carotenoid content in Chinese cabbage. Error bars indicate standard deviations. Different small letters on the bars indicate significant differences among treatments at *p* < 0.05.

The contents of chlorophyll a, chlorophyll b, and carotenoids in Chinese cabbage were significantly affected by chromium stress and PF-ZrO_2_ NP treatment (Figure 6). Chromium exposure (T2 and T4) resulted in 13.14% and 22.67% reductions in chlorophyll a, respectively, compared to the control (T1), indicating Cr-induced oxidative stress and degradation of photosynthetic pigments. Similarly, chlorophyll b content decreased by 24.45% and 30.56% in T2 and T4, respectively, showing that Cr toxicity disrupts pigment synthesis and chloroplast function. Carotenoid levels also showed a significant decline, with 30.73% and 49.86% reductions in T2 and T4, respectively, further confirming Cr-induced damage to light-harvesting pigments. The observed decrease in chlorophyll content under Cr stress is likely due to Cr contamination hindering crucial plant functions like photosynthesis and respiration, leading to reduced energy output, oxidative stress, and impaired nutrient uptake [9]. The decrease in chlorophyll content is a common response to Cr toxicity, as seen in mung bean seedlings where chlorophyll contents decreased by 57.09% under 50 µM Cr stress [9]. Similarly, in sweet potato plants, higher levels of Cr treatment resulted in significant reductions in photosynthetic attributes and chlorophyll content [73].

However, the application of PF-ZrO_2_ NPs (T3 and T5) improved pigment retention. Compared to T2, T3 showed a 5.41% increase in chlorophyll a, a 22.69% increase in chlorophyll b, and a 35.79% increase in carotenoid content, indicating the nanoparticles’ protective effects against Cr toxicity. Similarly, T5 (100 mg/kg Cr + 100 mg/kg PF-ZrO_2_ NPs) showed a 14.41% increase in chlorophyll a, 10.35% in chlorophyll b, and 53.95% in carotenoids compared to T4, highlighting the role of PF-ZrO_2_ NPs in mitigating Cr-induced pigment degradation. The positive effects of PF-ZrO_2_ NPs on pigment stabilization may be attributed to several factors. Firstly, PF-ZrO_2_ NPs may reduce Cr bioavailability in soil, limiting Cr uptake and accumulation in plant tissues [74]. Secondly, nanoparticles are known to enhance antioxidant enzyme activity, reducing ROS-induced damage to photosynthetic pigments [75]. Additionally, nanoparticles can interact with cellular components, stabilizing membrane integrity and improving nutrient uptake, thereby supporting better chloroplast function and pigment synthesis [76]. This suggests that green-synthesized PF-ZrO_2_ NPs could serve as an effective strategy to counteract heavy metal toxicity in plants, promoting healthier growth and improved photosynthetic efficiency.

### 3.10. Biochemical Responses of Chinese Cabbage Under Chromium Stress and PF-ZrO_2_ NP Application

Chromium (Cr) contamination significantly influenced the biochemical responses in the shoots and roots of Chinese cabbage (Figure S3). Exposure to chromium alone (T2 and T4) negatively affected plant physiology, leading to a decrease in shoot-soluble protein content by approximately 19.82% and 41.84%, respectively, compared to the control (T1). Root-soluble protein also markedly decreased under Cr stress, with reductions of 17.01% and 14.42% in T2 and T4. These results are in line with many studies. In Gautam et al. (2020) [77], it was observed that Cr treatment resulted in a decline of root length, shoot length, fresh weight, and dry weight in *Vigna radiata* seedlings. While not explicitly mentioning root-soluble protein, the study noted a general decrease in plant growth parameters under Cr stress. Fu et al. (2023) [27] reported that the addition of silicon (Si) and selenium (Se) to Chinese cabbage under Cr stress resulted in a 29.58% increase in soluble protein content. This implies that Cr stress alone had reduced the soluble protein content, which was then ameliorated by Si and Se treatment. However, nanoparticle treatments (T3 and T5) effectively counteracted these negative impacts, improving shoot-soluble protein by 9.63% and 14.70% and root protein content by 17.01% and 18.99%, respectively, when compared to Cr-only treatments. The increased protein content with PF-ZrO_2_ NP application likely reflects enhanced nitrogen metabolism and protein synthesis in response to reduced chromium toxicity.

Soluble sugar levels were similarly affected, with chromium-only treatments (T2 and T4) causing significant reductions (9.64% and 87.91% in the shoots and substantial reductions in the roots) (Figure S3). At low levels of Cr exposure (25 μM), studies have shown an increase in soluble sugar content in sweet potato plants [73]. This increase in sugar levels may be part of the plant’s initial stress response mechanism. However, at higher Cr concentrations (100–200 μM), a significant reduction in soluble sugar content was observed, likely due to the severe oxidative damage and reduced photosynthetic activity caused by Cr toxicity [73]. Conversely, PF-ZrO_2_ NPs significantly increased soluble sugar content in the shoots by 36.72% (T3 vs. T2) and 86.02% in the roots (T5 vs. T4). This improved sugar accumulation under nanoparticle treatment indicates better carbohydrate metabolism, possibly supporting osmotic regulation and energy supply under stress conditions. Several studies have demonstrated this beneficial impact across different plant species and stress types. Nanoparticles were found to enhance soluble sugar content in drought-stressed canola plants, contributing to osmotic potential regulation and improved stress tolerance [78]. These findings suggest that nanoparticles can positively influence sugar accumulation and energy metabolism to help plants cope with abiotic stresses.

Proline content increased significantly under chromium stress as a defensive response. Shoot proline increased by 24.46% and 44.96% in T2 and T4 compared to control plants, while root proline increased by 27.65% and 66.16%, respectively. However, PF-ZrO_2_ NP application effectively moderated proline levels by 10.54% in shoots (T3) and 18.99% in T5, indicating that nanoparticles helped mitigate chromium-induced stress, stabilizing plant physiological conditions. Nanoparticles (NPs) have shown significant potential in mitigating chromium-induced stress and stabilizing plant physiological conditions. NPs, when applied to rice plants grown in a chromium-saturated medium, improved plant growth, biomass, yield, and photosynthetic activity by enhancing chlorophyll contents and alleviating oxidative damage [63]. The application of NPs reduced the uptake and accumulation of Cr in plants by increasing the bioavailability of micronutrients, thereby decreasing oxidative damage and enhancing enzymatic and non-enzymatic activity to withstand Cr stress [63].

These observations suggest PF-ZrO_2_ NPs play an important role in alleviating chromium-induced stress by enhancing protective biochemical mechanisms in plants, consistent with previous findings that engineered nanoparticles improve plant resilience by reducing oxidative stress and improving nutrient metabolism under heavy metal exposure.

### 3.11. Antioxidant Enzyme Activities Under Chromium Stress and PF-ZrO_2_ NP Application

**Figure 7:**
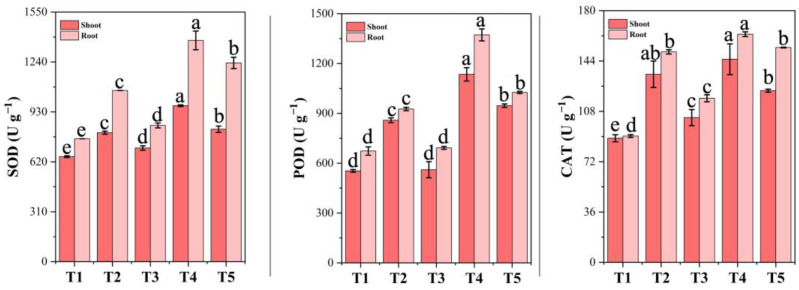
Effect of chromium (Cr) stress and PF-ZrO_2_ NP application on superoxide dismutase (SOD), peroxidase (POD), and catalase (CAT) activities in shoots and roots of Chinese cabbage. Error bars indicate standard deviations. Different small letters on the bars indicate significant differences among treatments at *p* < 0.05.

The activities of superoxide dismutase (SOD), peroxidase (POD), and catalase (CAT) in both the shoots and roots of Chinese cabbage were significantly influenced by chromium stress and PF-ZrO_2_ NP treatment (Figure 7). Chromium exposure (T2 and T4) resulted in a substantial increase in antioxidant enzyme activity, reflecting a plant defense mechanism against Cr-induced oxidative stress. In the shoots, SOD activity increased by 22.82% in T2 and 48.55% in T4 compared to the control (T1), indicating that plants activated SOD to neutralize superoxide radicals generated under Cr stress. SOD acts as the first line of defense in the plant antioxidant system, effectively eliminating reactive oxygen species (ROS) under harsh environmental conditions [79]. It dismutates superoxide anion free radicals to generate hydrogen peroxide and molecular oxygen, shielding cells from oxidative injury [80]. However, when PF-ZrO_2_ NPs were applied (T3 and T5), SOD activity showed a 11.88% and 15.12% increase compared to T2 and T4, respectively, suggesting that nanoparticles helped further enhance the antioxidant response, aiding in Cr detoxification [81]. In the roots, T2 and T4 exhibited 39.31% and 80.07% increases, respectively, while T3 and T5 improved SOD activity by 20.41% and 10.20% over their respective Cr-only treatments, demonstrating the role of nanoparticles in maintaining oxidative homeostasis. Gold nanoparticles conjugated with transferrin were found to downregulate major proteins associated with the glutathione-thioredoxin antioxidant pathway in prostate cancer cells, inducing an oxidative stress response [82].

Similarly, POD activity increased significantly under Cr stress, with T2 and T4 showing 54.98% and 104.95% increases in the shoots and 37.58% and 103.89% increases in the roots, respectively. This suggests that Cr stress induced higher hydrogen peroxide (H_2_O_2_) levels, requiring an enhanced POD response to break down excess ROS [83]. This increase in ROS production, particularly H_2_O_2_, is a common response to various abiotic stresses, including heavy metal toxicity. To combat this oxidative stress, plants activate their antioxidant defense systems, which include enzymatic antioxidants like peroxidases [84]. PF-ZrO_2_ NP-treated plants (T3 and T5) showed a 34.66% and 16.63% increase in POD activity in the shoots, and a 25.27% and 25.28% increase in the roots, respectively, compared to T2 and T4. This improvement suggests that nanoparticles supported the plant’s enzymatic detoxification mechanism, reducing oxidative stress more effectively [85].

The activity of CAT, a key enzyme involved in H_2_O_2_ detoxification, also followed a similar pattern. T2 and T4 showed a 51.55% and 63.65% increase in the shoots, and a 66.68% and 80.65% increase in the roots, respectively, compared to T1. This highlights the severity of Cr-induced oxidative stress, leading to excessive H_2_O_2_ accumulation. Oxidative stress is characterized by an imbalance between the production of reactive oxygen species (ROS) and the body’s ability to counteract their harmful effects through neutralization by antioxidants [86]. Excessive ROS accumulation, including H_2_O_2_, can lead to cellular damage and contribute to various pathological conditions [87]. PF-ZrO_2_ NP treatment (T3 and T5) enhanced CAT activity by 23.02% and 15.47% in the shoots, and by 22.04% and 5.84% in the roots compared to T2 and T4, respectively. This suggests that nanoparticles helped regulate H_2_O_2_ levels and protect cellular components from oxidative damage.

The enhanced antioxidant enzyme activities in T3 and T5 demonstrate that PF-ZrO_2_ NPs effectively alleviated Cr-induced oxidative stress by stabilizing antioxidant defenses, reducing ROS accumulation, and maintaining enzymatic homeostasis. Nanoparticles have been shown to mitigate the negative impacts of various abiotic stresses, including heavy metal toxicity, in plants [88]. In the case of pea plants under drought stress, boron oxide nanoparticles stimulated the activity of antioxidant enzymes such as APX, SOD, GPX, and CAT, effectively reducing oxidative stress markers like MDA and H_2_O_2_ [89].

### 3.12. Plant Growth Parameters Under Chromium Stress and PF-ZrO_2_ NP Application

The shoot and root biomass as well as the shoot and root length of Chinese cabbage were significantly affected by chromium stress and PF-ZrO_2_ NP treatment (Figure S4). Chromium contamination (T2 and T4) resulted in a substantial reduction in plant growth, reflecting Cr-induced toxicity and its negative impact on plant metabolism and nutrient uptake. Shoot biomass decreased by 23.72% and 39.61% in T2 and T4, respectively, compared to the control (T1), while root biomass declined by 49.70% and 65.72% in the respective treatments. This reduction suggests that Cr stress severely affected biomass accumulation, likely due to impaired nutrient uptake, oxidative stress, and growth inhibition [90]. It also impairs nutrient uptake and allocation, leading to decreased plant biomass [91]. For instance, in sweet potato plants, higher levels of Cr treatment resulted in significant deleterious effects on growth, biomass, and photosynthetic attributes [73]. The negative effects were more pronounced in T4, indicating that higher Cr concentrations (100 mg/kg) resulted in greater biomass suppression.

However, the application of PF-ZrO_2_ NPs (T3 and T5) significantly improved biomass production. T3 exhibited a 24.53% increase in shoot biomass and a 34.61% increase in root biomass compared to T2, while T5 showed a 32.03% and 59.25% increase in shoot and root biomass compared to T4, respectively. These improvements suggest that PF-ZrO_2_ NPs mitigated Cr toxicity by reducing Cr bioavailability, improving nutrient uptake, and enhancing antioxidant defense mechanisms. Several studies have demonstrated that nanoparticles can help mitigate chromium toxicity in plants by reducing Cr bioavailability, improving nutrient uptake, and enhancing antioxidant defense mechanisms. For instance, iron nanoparticles (Fe NPs) significantly improved plant growth, biomass, yield, and photosynthetic activity in rice plants under Cr stress [63]. Fe NPs reduced Cr uptake and accumulation in plants by increasing the bioavailability of micronutrients and enhancing enzymatic and non-enzymatic antioxidant activity [63].

Similarly, the shoot and root lengths were significantly reduced under Cr stress. T2 and T4 showed a 15.60% and 25.28% decrease in shoot length, while root length was reduced by 12.17% and 19.60%, respectively, compared to T1. This reduction in plant height and root growth suggests that Cr exposure hindered cell elongation and division, leading to stunted growth. In line with our study, the authors of [9] reported that Cr toxicity causes severe damage to mung bean seedlings, limiting their growth and physiological characteristics. Similarly, in sweet potato plants, higher levels of Cr treatment led to significant deleterious effects on growth and biomass [73].

Upon PF-ZrO_2_ NP application, T3 and T5 exhibited significant improvements in plant length. T3 showed a 14.69% increase in shoot length and a 5.41% increase in root length compared to T2, while T5 exhibited a 13.28% and 6.90% increase in shoot and root length compared to T4, respectively. These findings suggest that PF-ZrO_2_ NPs alleviated Cr toxicity by improving soil conditions, enhancing root development, and supporting shoot elongation. Propping up our study, several pieces of research have shown that metal oxide nanoparticles can alleviate chromium toxicity in plants by improving growth parameters and reducing oxidative stress. For example, Yadav et al. (2024) [74] and Basit et al. (2022) [92] found that nanoparticles can enhance plant growth, biomass accumulation, and photosynthetic activity in plants under Cr stress. These nanoparticles also reduced Cr uptake and accumulation in plant tissues while improving nutrient acquisition.

### 3.13. Chromium Accumulation in Plant Tissues Under PF-ZrO_2_ NP Application

**Figure 8:**
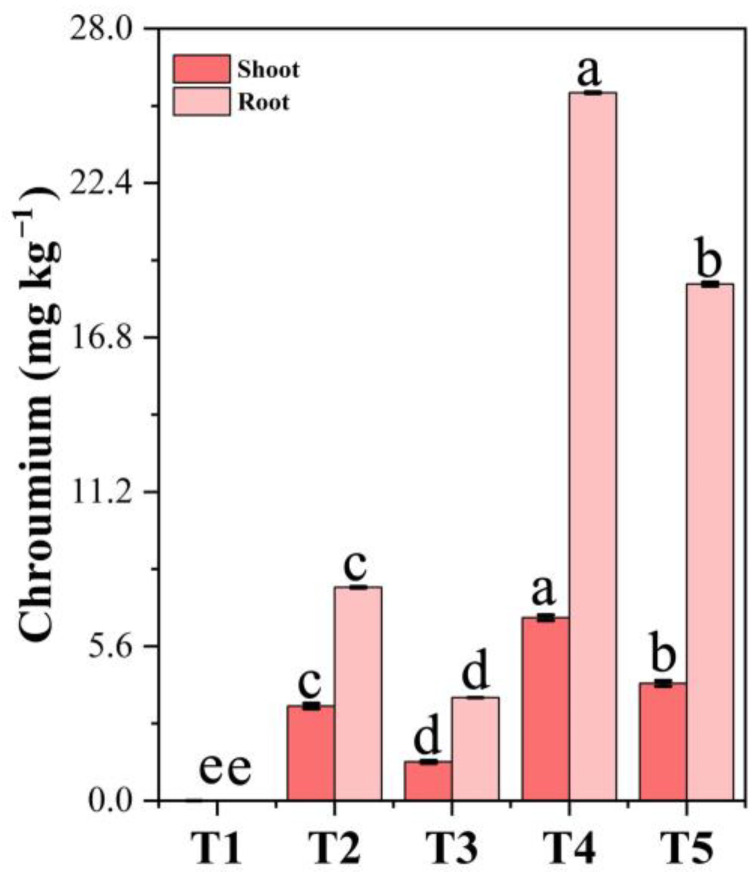
Effect of chromium (Cr) stress and PF-ZrO_2_ NP application on chromium accumulation (mg/kg) in shoots and roots of Chinese cabbage. Error bars indicate standard deviations. Different small letters on the bars indicate significant differences among treatments at *p* < 0.05.

Chromium stress significantly increased Cr accumulation in both the shoots and roots of Chinese cabbage, with the roots showing higher concentrations than the shoots (Figure 8). This pattern reflects the typical plant response of restricting heavy metal translocation to aerial parts as a protective mechanism [73].

However, PF-ZrO_2_ NP application significantly reduced Cr accumulation in both plant parts. T3 (50 mg/kg Cr + 100 mg/kg PF-ZrO_2_ NPs) showed a 1.59-fold decrease in shoot Cr and a 1.52-fold reduction in root Cr compared to T2, while T5 (100 mg/kg Cr + 100 mg/kg PF-ZrO_2_ NPs) exhibited a 1.36-fold and 1.27-fold reduction in shoot and root Cr, respectively, compared to T4. These reductions suggest that PF-ZrO_2_ NPs effectively immobilized Cr in soil, reducing its bioavailability and uptake by plant roots. The greater Cr reduction in T3 compared to T5 suggests that PF-ZrO_2_ NPs were more effective at lower Cr concentrations, likely due to higher adsorption efficiency and reduced metal stress at moderate contamination levels. At lower Cr concentrations, several studies demonstrate higher adsorption efficiency and reduced metal stress. Li et al. (2023) [93] report that the adsorption effect of nanoparticles is optimal when the initial Cr(VI) concentration is 40 mg/L. This suggests that the adsorbent performs better at lower Cr concentrations.

### 3.14. Macro and Micronutrient Uptake Under Chromium Stress and PF-ZrO_2_ NP Application

The uptake of nitrogen (N), phosphorus (P), potassium (K), calcium (Ca), and magnesium (Mg) in the shoots and roots of Chinese cabbage was significantly influenced by chromium stress and PF-ZrO_2_ NP treatment (Figure S5). Chromium exposure (T2 and T4) led to a substantial reduction in macronutrient accumulation, likely due to Cr interference with ion transport, competition for uptake sites, and the disruption of membrane permeability. In the shoots, T2 and T4 showed 20.62% and 35.89% reductions in nitrogen content, respectively, compared to the control (T1), while root N decreased by 20.62% in T2 and 35.89% in T4, confirming Cr-induced nutrient stress. Similarly, phosphorus levels declined by 26.77% and 44.09% in the shoots and 26.75% and 44.07% in the roots, respectively, under Cr stress, indicating that Cr toxicity hindered P translocation. Potassium, an essential macronutrient for enzyme activation and osmotic regulation, was reduced by 25.62% and 44.20% in the shoots and 25.61% and 44.21% in the roots, respectively, in T2 and T4, suggesting Cr-induced inhibition of K+ uptake and disruption of stomatal regulation. Calcium (Ca) and magnesium (Mg) levels also followed a decreasing trend, with T2 and T4 exhibiting 40.80% and 53.30% reductions in shoot Ca and 44.96% and 56.35% in root Ca, respectively. Magnesium, a crucial component of chlorophyll synthesis, was 28.85% and 41.91% lower in shoots and 37.02% and 44.99% lower in roots in T2 and T4, respectively. These findings suggest that chromium (Cr) stress significantly impairs plant mineral nutrition, leading to deficiencies that negatively affect photosynthesis, enzyme function, and overall plant growth. Multiple studies have demonstrated the detrimental effects of Cr on various plant species and their physiological processes. Cr stress has been shown to inhibit the uptake and accumulation of essential nutrients in plants. For instance, in chickpea plants, Cr stress significantly reduced mineral acquisition, which directly impacted photosynthetic pigments and gas exchange traits [94]. Similarly, in mung bean seedlings, Cr toxicity resulted in decreased chlorophyll content and reduced crop growth rate [9].

However, the application of PF-ZrO_2_ NPs (T3 and T5) significantly improved macronutrient uptake. T3 exhibited a 16.83% increase in shoot N and a 16.83% increase in root N compared to T2, suggesting that nanoparticles enhanced nitrogen assimilation and transport. Similarly, phosphorus uptake increased by 21.04% in shoots and 21.05% in roots, while potassium content increased by 18.83% in shoots and 18.81% in roots, confirming that PF-ZrO_2_ NPs facilitated macronutrient retention, mitigating Cr-induced nutrient loss. The positive effects of PF-ZrO_2_ NPs on Ca and Mg retention were also evident. Compared to T2, T3 showed a 33.64% increase in shoot Ca and 52.81% in root Ca, while Mg content improved by 15.25% in shoots and 37.41% in roots, demonstrating that nanoparticles improved cation exchange capacity, stabilized soil structure, and enhanced nutrient bioavailability. T5 (higher Cr concentration) showed a 58.16% and 50.59% increase in shoot and root Ca and a 23.91% and 39.43% increase in shoot and root Mg compared to T4, indicating that PF-ZrO_2_ NPs played a critical role in maintaining mineral balance under Cr stress. The findings align with previous research on the use of nanoparticles for improving nutrient uptake efficiency and mitigating heavy metal contamination in soils. Several studies support the potential of nanoparticles as sustainable soil amendments for enhancing plant health in contaminated environments. Nanoparticles (NPs) have been shown to enhance corn growth, nutrient uptake, and nutrient use efficiency at optimal application rates [95]. The improved plant performance was attributed to the increased availability of phosphorus and nitrogen in amended soils. Similarly, nanomaterials have demonstrated promise in promoting crop protection, enhancing productivity, and reducing contamination in agricultural settings [96].

### 3.15. Oxidative Stress Indicators Under Chromium Stress and PF-ZrO_2_ NP Application

**Figure 9:**
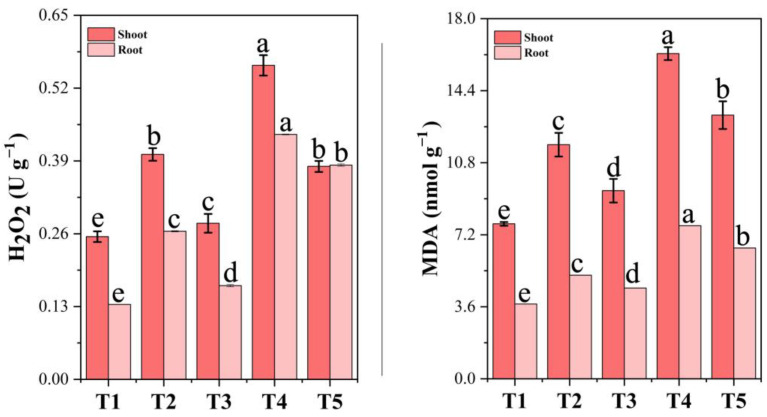
Effect of chromium (Cr) stress and PF-ZrO_2_ NP application on hydrogen peroxide (H_2_O_2_) and malondialdehyde (MDA) levels in shoots and roots of Chinese cabbage. Error bars indicate standard deviations. Different small letters on the bars indicate significant differences among treatments at *p* < 0.05.

The levels of hydrogen peroxide (H_2_O_2_) and malondialdehyde (MDA) in both the shoots and roots of Chinese cabbage were significantly influenced by chromium stress and PF-ZrO_2_ NP application (Figure 9). Chromium exposure (T2 and T4) resulted in a substantial increase in H_2_O_2_ and MDA levels, indicating severe oxidative stress.

In the shoots, H_2_O_2_ levels increased by 57.65% and 120.05% in T2 and T4, respectively, compared to the control (T1), suggesting that Cr exposure led to excessive reactive oxygen species (ROS) production [97]. A similar trend was observed in the roots, where T2 and T4 exhibited a 97.83% and 227.08% increase in H_2_O_2_, respectively, compared to T1. Oxidative stress occurs when there is an imbalance between the production of reactive oxygen species (ROS) and the antioxidant defense systems [98]. In this context, a sharp rise in H_2_O_2_ under higher Cr concentrations (T4) suggest a severe disruption of cellular redox homeostasis, potentially leading to oxidative damage and impaired metabolic functions.

However, the application of PF-ZrO_2_ NPs (T3 and T5) significantly reduced H_2_O_2_ accumulation compared to their respective Cr-only treatments (T2 and T4). T3 showed a 30.62% reduction in the shoots and a 36.75% reduction in the roots compared to T2, while T5 exhibited a 32.17% and 12.51% decrease compared to T4. This indicates that PF-ZrO_2_ NPs helped mitigate oxidative stress by reducing ROS accumulation, possibly by enhancing antioxidant enzyme activity and stabilizing cellular membranes. In line with our study, several studies have demonstrated that certain nanoparticles can help mitigate oxidative stress by reducing reactive oxygen species (ROS) accumulation and enhancing antioxidant enzyme activity. For example, Soni et al. (2022) [99] mention that TiO_2_, Ag, and Si nanoparticles can reduce UV-B stress in plants by reducing oxidative stress by mimicking antioxidants or improving antioxidant enzyme activities. Similarly, Ou et al. (2021) [100] describe a hydrogel containing polydopamine nanoparticles that can improve the activity of superoxide dismutase and glutathione peroxidase and reduce the levels of ROS and malondialdehyde, thus preventing oxidative damage to cells.

MDA, a key indicator of lipid peroxidation and membrane damage, followed a similar trend. Chromium exposure led to 51.07% and 109.81% increases in MDA levels in the shoots of T2 and T4, respectively, while in the roots, the increase was 38.54% in T2 and an extreme 204.72% in T4, highlighting severe Cr-induced oxidative membrane damage.

However, PF-ZrO_2_ NPs significantly reduced MDA content, with T3 showing a 19.68% reduction in the shoots and a 12.35% reduction in the roots compared to T2, while T5 exhibited an 18.91% and 99.91% decrease compared to T4, respectively. These results suggest that PF-ZrO_2_ NPs helped prevent lipid peroxidation and improved membrane integrity, reducing Cr-induced oxidative damage. The enhanced antioxidant response in T3 and T5 suggests that PF-ZrO_2_ NPs improved ROS scavenging mechanisms, protecting plant cells from oxidative stress and maintaining physiological stability. Several studies have demonstrated that various nanoparticles have been shown to possess ROS-scavenging capabilities. Wu et al. (2023) [101] describe nanoparticles exhibiting a significant ROS-scavenging capability, enhancing therapeutic effects in an atopic dermatitis mouse model. Similarly, Nag et al. (2022) [102] report that nanoparticles showed enhanced cellular uptake and efficient ROS-scavenging activity in live cells, protecting them from oxidative stress.

## 4. Conclusions

This study confirms the effectiveness of green-synthesized zirconia nanoparticles (PF-ZrO_2_ NPs), derived from *Sonchus asper* extract, in mitigating chromium (Cr) toxicity in soil–plant systems. The application of PF-ZrO_2_ NPs not only significantly reduced Cr bioavailability and translocation to Chinese cabbage but also alleviated oxidative stress and promoted plant growth. These outcomes were accompanied by improved soil physicochemical properties, suggesting that PF-ZrO_2_ NPs contribute to overall soil health restoration.

Structurally, PF-ZrO_2_ NPs exhibit favorable features, such as a high surface area, mesoporous structure, and functional surface groups that enhance their adsorption capacity and facilitate Cr immobilization. Moreover, the incorporation of plant-derived carbon (~17.6%) represents a major distinction from conventionally synthesized zirconia. This bio-organic component may act synergistically with the inorganic phase, potentially enhancing sorption performance, modulating microbial activity, and improving soil fertility mechanisms comparable to the beneficial roles attributed to biochar in soil amendment.

From a practical standpoint, although the current application rate (500 mg/kg dry soil) may raise concerns regarding large-scale feasibility, the green synthesis strategy employed here offers notable advantages. *Sonchus asper*, a fast-growing and widely available plant, provides a renewable and low-cost biomass source for nanoparticle fabrication. This enhances the economic viability of the method and supports its potential for field-scale implementation. Furthermore, the integration of plant-derived materials into nanoparticle synthesis aligns well with the principles of circular bioeconomy and sustainable land management, promoting resource recycling and environmentally responsible remediation practices.

Future studies should aim to assess the long-term stability and environmental safety of PF-ZrO_2_ NPs under diverse soil conditions, explore cost-effective upscaling strategies, and investigate synergistic effects with organic matter, microbial inoculants, or other remediation materials. Field-scale validation will be essential to fully realize the potential of PF-ZrO_2_ NPs as a sustainable and multifunctional amendment for remediating Cr-contaminated soils and promoting safe crop production.

## Data Availability

This paper and its Supplementary Information contain all relevant data.

## Supplementary Materials

The following supporting information can be downloaded at: https://www.mdpi.com/article/10.3390/toxics13050324/s1, Figure S1. (A) Energy-dispersive X-ray spectroscopy (EDS) spectrum of PF-ZrO_2_ nanoparticles, confirming the presence of zirconium (Zr), oxygen (O), and carbon (C) as primary elements, with their respective atomic percentages. (B) Elemental mapping images showing the uniform distribution of Zr, O, and C, indicating the homogeneity and purity of the synthesized nanoparticles; Figure S2. Effect of chromium (Cr) stress and PF-ZrO_2_ NP application on soil phosphorus (P), potassium (K), nitrogen (N), magnesium (Mg), and calcium (Ca) contents. T1: Control (0 mg/kg Cr + 0 mg/kg PF-ZrO_2_ NPs), T2: 50 mg/kg Cr, T3: 50 mg/kg Cr + 500 mg/kg PF-ZrO_2_ NPs, T4: 100 mg/kg Cr, T5: 100 mg/kg Cr + 500 mg/kg PF-ZrO_2_ NPs. Results are the mean values ± standard deviation (*n* = 3). Error bars indicate standard deviations. Different small letters on the bars indicate significant differences among treatments at *p* < 0.05; Figure S3. Effect of chromium (Cr) stress and PF-ZrO_2_ NP application on soluble protein, soluble sugar, and proline content in the shoots and roots of Chinese cabbage; Figure S4. Effect of chromium (Cr) stress and PF-ZrO_2_ NP application on shoot and root length (cm) and biomass (g) of Chinese cabbage; Figure S5. Effect of chromium (Cr) stress and PF-ZrO_2_ NP application on magnesium (Mg), potassium (K), phosphorus (P), calcium (Ca), and nitrogen (N) content in shoots and roots of Chinese cabbage.
