# Supplementary material for: Eco-Friendly Synthesis of Zirconia Nanoparticles Using Sonchus asper Extract: A Sustainable Approach to Enhancing Chinese Cabbage Growth and Remediating Chromium-Contaminated Soil
Source: Toxics. 2025 Apr 22;13(5):324. doi: 10.3390/toxics13050324 (PMC12115994; doi:10.3390/toxics13050324)
Supplement: Supplementary file 1 [file toxics-13-00324-s001.zip › toxics-3556756-supplementary.pdf]

Supplementary Information

Eco-friendly synthesis of zirconia nanoparticles using *Sonchus asper* extract: a sustainable approach to enhancing Chinese cabbage growth and remediating chromium-contaminated soil

Guojie Weng[1,2#], Weidong Li[1,2#], Fengyue Qin[1,2], Menglu Dong[1,2], Shuangqi Yue[1,2], Sajid Mehmood[1,2*], Xu Wang[1,2*]

[1]Center for Eco-Environment Restoration of Hainan Province, School of Ecology,

Hainan University, Haikou, 570228, China

[2]School of Topical Agriculture and Foresty, Hainan University, Haikou, 570228, China

#These authors contributed equally to this paper

***Corresponding author**

Dr. Sajid Mehmood: E-mail: drsajid@hainanu.edu.cn; **Dr. Xu Wang:** Email: 990991@hainanu.edu.cn

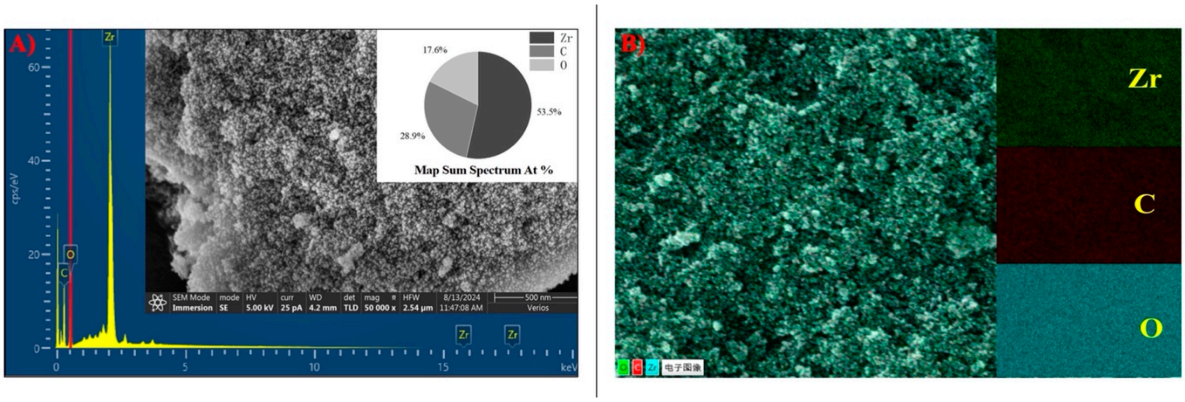

Figure S1. (A) Energy-dispersive X-ray spectroscopy (EDS) spectrum of PF-ZrO_2 nanoparticles, confirming the presence of zirconium (Zr), oxygen (O), and carbon (C) as primary elements, with their respective atomic percentages. (B) Elemental mapping images showing the uniform distribution of Zr, O, and C, indicating the homogeneity and purity of the synthesized nanoparticles.

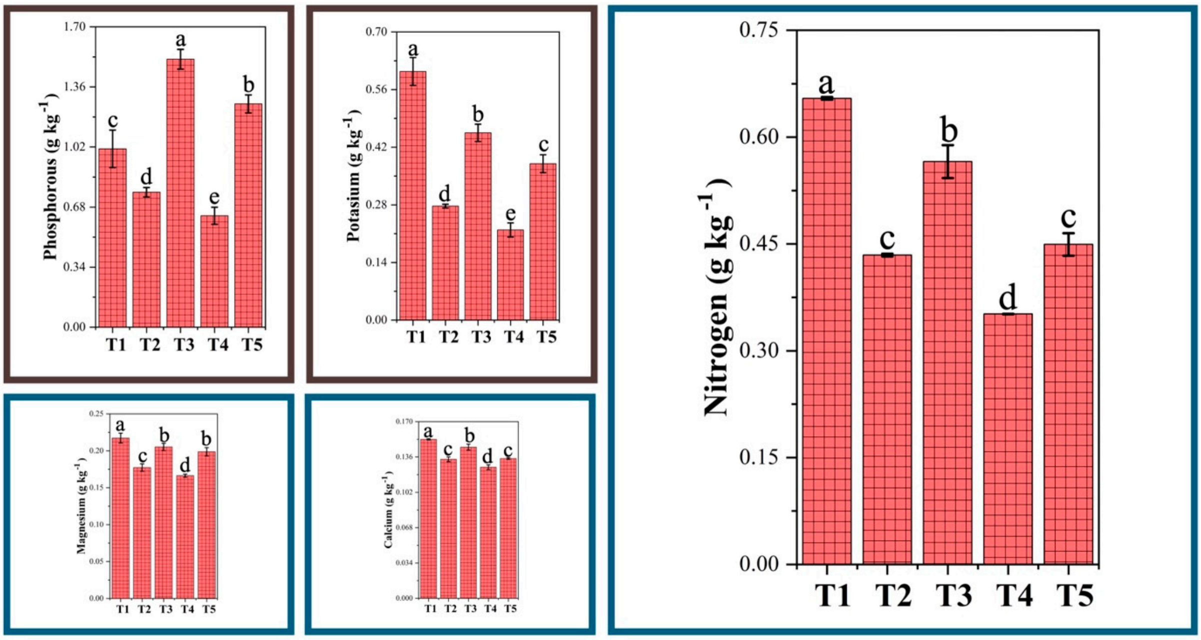

Figure S2. Effect of chromium (Cr) stress and PF-ZrO_2 NP application on soil phosphorus (P), potassium (K), nitrogen (N), magnesium (Mg), and calcium (Ca) contents. T1: Control (0 mg/kg Cr + 0 mg/kg PF-ZrO_2 NPs), T2: 50 mg/kg Cr, T3: 50 mg/kg Cr + 500 mg/kg PF-ZrO_2 NPs, T4: 100 mg/kg Cr, T5: 100 mg/kg Cr + 500 mg/kg PF-ZrO_2 NPs. Results are the mean values ± standard deviation (n = 3). Error bars indicate standard deviations. Different small letters on the bars indicate significant differences among treatments at $P<0.05$.

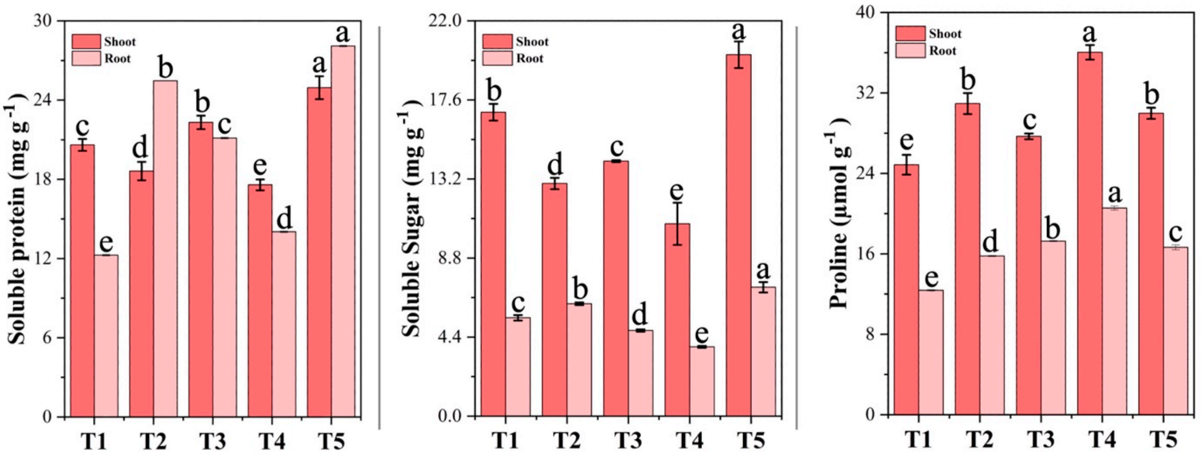

Figure S3. Effect of chromium (Cr) stress and PF-ZrO_2 NP application on soluble protein, soluble sugar, and proline content in the shoots and roots of Chinese cabbage.

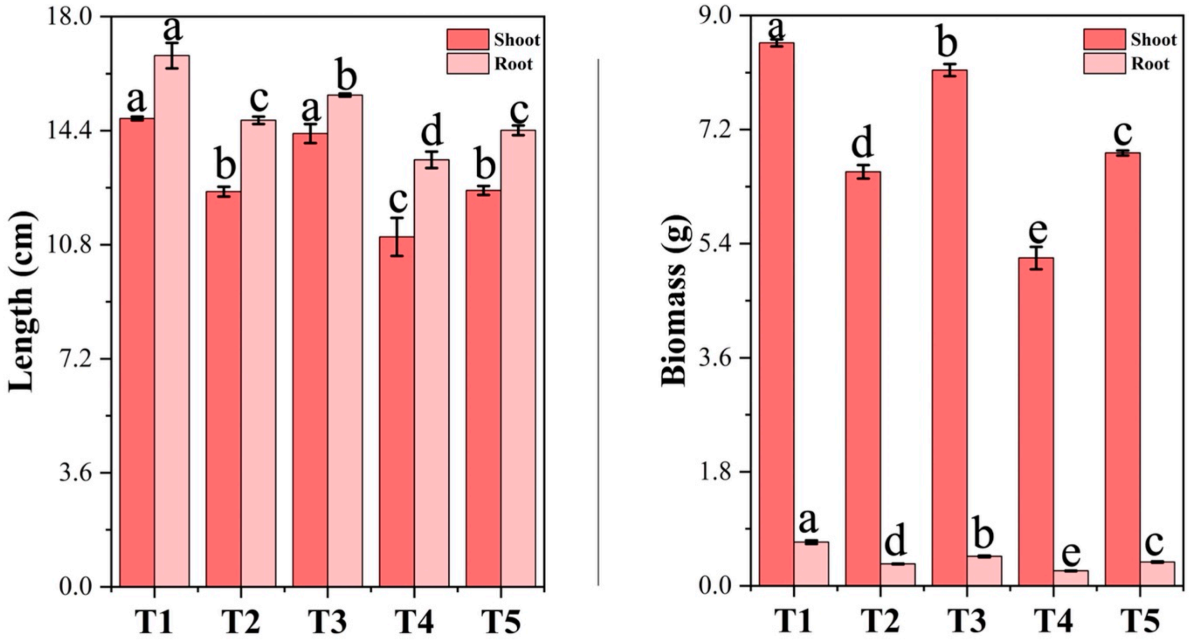

Figure S4. Effect of chromium (Cr) stress and PF-ZrO_2 NP application on shoot and root length (cm) and biomass (g) of Chinese cabbage.

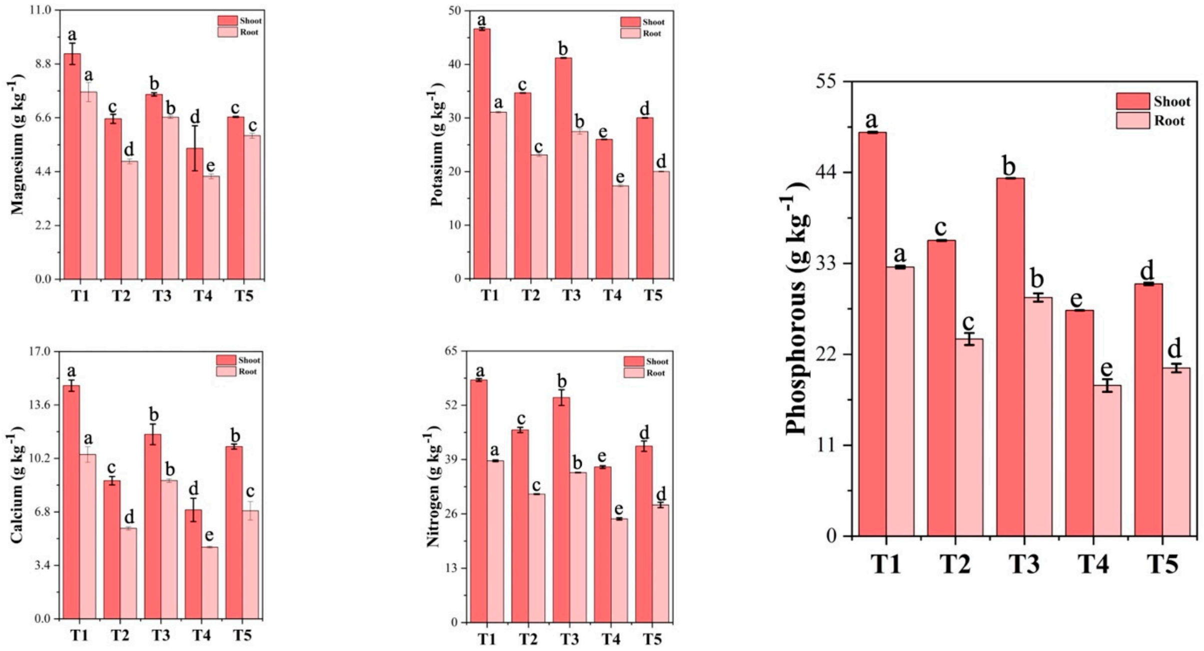

Figure S5. Effect of chromium (Cr) stress and PF-ZrO_2 NP application on magnesium (Mg), potassium (K), phosphorus (P), calcium (Ca), and nitrogen (N) content in shoots and roots of Chinese cabbage.
